# Systematic review of EASY-care needs assessment for community-dwelling older people

**DOI:** 10.1093/ageing/afv050

**Published:** 2015-04-24

**Authors:** Christopher Craig, Neil Chadborn, Gina Sands, Helena Tuomainen, John Gladman

**Affiliations:** 1 National Institute for Health Research, CLAHRC East Midlands, University of Nottingham, Nottingham, UK; 2 Warwick Medical School, Division of Mental Health and Well-being, University of Warwick, Warwick, UK

**Keywords:** EASY-Care, geriatric assessment, needs assessment, systematic review, older people

## Abstract

**Background:** undertaking comprehensive geriatric assessments (CGAs) combined with long-term health and social care management can improve the quality of life of older people [ 1]. The EASY-Care tool is a CGA instrument designed for assessing the physical, mental and social functioning and unmet health and social needs of older people in community settings or primary care. It has also been used as a frailty assessment tool and for gathering population-level data.

**Objective:** to review the evidence of reliability, validity and acceptability of EASY-Care and its appropriateness for assessing the needs of community-dwelling older people.

**Methods:** systematic search of literature databases using pre-defined search terms (January 1994—May 2014) for English language articles reporting on the reliability, validity, acceptability and implementation of EASY-Care in primary care and community settings. Eligible articles were critically reviewed. Discussion papers mapping professionals' use of the tool were also included as these could be considered an aspect of validity.

**Results:** twenty-nine papers met the inclusion criteria and underwent data extraction. A narrative synthesis was performed, because there was a variety of quantitative and qualitative outcomes and characteristics. Reliability evidence for EASY-Care is minimal. Evidence for validity is good, and it has received numerous positive endorsements of acceptability in international settings from older people and practitioners.

**Conclusion:** evidence supports the use of EASY-Care for individual needs assessment; further research is needed for other uses. Of the papers that made statements about who should administer EASY-Care, the majority indicated that nurses were preferable to self-completion.

## Introduction

With older age comes an increased chance of frailty constituted by physical, social, mental and possibly environmental factors [2]. Complex interventions have been shown to help older people live safely and independently, and can be tailored to meet individuals' needs and preferences through personalised assessments [1]. A tool to do this should ideally be comprehensive, covering a broad range of domains; person-centred, putting the older person at the heart of the assessment; proven to be valid and reliable for clinical use; acceptable for both patients and practitioners to use internationally; and informative to local health and social care commissioners to assist in health resource planning.

One example of a needs assessment tool is ‘EASY-Care’ which was developed in the United Kingdom, United States and Europe [3]. The first version from 1994, ‘EASY’ consisted of 31 questions. This was further refined in 1999, 2004 and 2010. The current version is a three-part questionnaire consisting of 49 core questions covering a broad range of physical, mental, social and environmental domains. EASY-Care incorporates questions from several validated and published health outcome measures including the Medical Outcome Scale Short Form 36, Barthel Index of Activities of Daily Living [4], the Duke Older Americans Resources and Services Instrumental Activities of Daily Living (Duke OARS IADL) [5] and items from a former World Health Organisation (WHO) multinational survey on the socio-medical status of elderly people [3]. A ‘not-for-profit’ company has been set up in the United Kingdom to host and licence the EASY-Care tool, the EASY-Care website provides comprehensive information about the assessment tool [6].

Several papers have commented on EASY-Care's development [3, 7, 8], but no systematic review analysing the potential benefits of using EASY-Care as a tool to support comprehensive geriatric assessment for community-dwelling older people has been published. This review seeks to examine the evidence of validity, reliability and acceptability of EASY-Care. We use ‘acceptability’ in this context as a broad term to incorporate effectiveness, cross-cultural acceptability to practitioners and older people, cost-effectiveness and feasibility. The main focus of this review is to scope available literature with empirical evidence; however, other reviews and commentaries are also included for completeness. This systematic review was outlined in the SOPRANO (Supporting Older People though Assessing Needs and Outcomes) study protocol which received scientific committee approval.

## Search strategy and selection criteria

A systematic search was carried out with the search terms of the key words ‘EASY-Care’ OR ‘EASYCare’ in the title or abstract for articles published from January 1994 until May 2014 (the original EASY-Care instrument was finalised in 1994), limited to humans. The following databases were searched:

- OVID MEDLINE
- OVID EMBASE
- CINAHL
- Web of Science
- Cochrane Library
- AGEINFO
- ASSIA: Applied Social Sciences Index and Abstracts
- The National Research Register (NRR) Archive
- NICHSR (National Information Center on Health Services Research and Health Care Technology
- NHS CRD DARE/HTA/EED (http://www.crd.york.ac.uk)

References from included articles from the database search were snowballed to ascertain other potentially eligible articles. Searches for ‘unpublished’ or ‘grey’ literature from several grey literature databases were undertaken to reveal any non-peer-reviewed articles which may have been relevant. Experts at EASY-Care (Judith Long, Project Officer at EASY-Care) also provided articles.

## Inclusion criteria

Titles and abstracts were screened for the term ‘EASY-Care’ by two reviewers, and then full articles were reviewed and included if they met the following criteria:

- Investigated the reliability, validity or acceptability of EASY-Care
  OR
  Reported on the implementation of EASY-Care within a complex intervention, such as setting, population, stakeholders, barriers or facilitators.
- The EASY-Care tool was administered on older people (50 years plus) based in community and/or primary care settings.
- Published in English.

Any disagreements about eligibility were resolved by discussion between the two reviewers.

## Results

**Figure 1.**
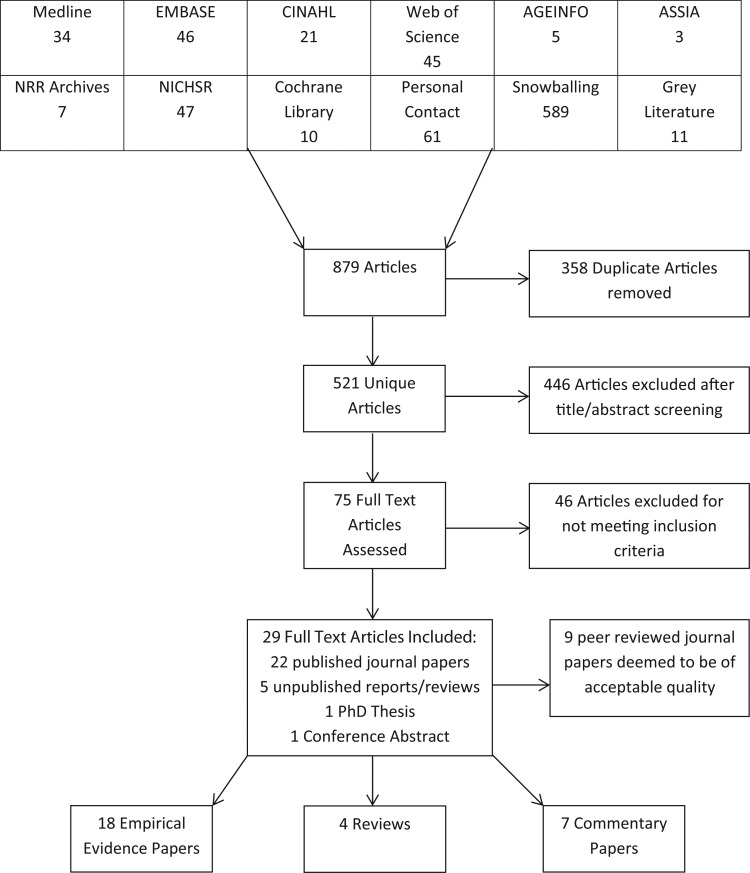
Flow diagram of selected articles included in review.

Eight hundred and seventy-nine articles were retrieved, of which 521 were screened once duplicates were removed (Figure 1). Through title and abstract screening, 446 articles did not meet the inclusion criteria and were excluded. The full text of the remaining 75 articles was assessed. Forty-six were excluded after examining the full text, leaving 29 articles for inclusion in the review in Table 1.

**Table 1.** Breakdown of publications included by type

Type of publication	List of authors and years of publication
Empirical Evidence	Bath *et al.* (2000) [12], Bath *et al.* (1998) [13], Faculty of Moscow (2008) [14], Fernandes *et al.* (2009) [15], Jerilu *et al.* (2013) [16], Keiren *et al.* (2013) [17], Lambert *et al.* (2007)^a^ [9], Lambert *et al.* (2007)^a^ [10], Lambert *et al.* (2009)^a^ [11], Melis *et al.* (2008)^b^ [18], Melis *et al.* (2008)^b^ [19]^b^, Msambichaka *et al.* (2014) [20], Philip *et al.* (2014) [21], Philp *et al.* (2002) [22], Philp *et al.* (2001)[23], van Kempen (2013)^c^ [24], van Kempen *et al.* (2012)^c^ [25], van Kempen *et al.* (2013)^c^ [26], van Kempen *et al.* (2014)^c^ [27].
Reviews	Foreman *et al.* (2004) [28], Haywood *et al.* (2004) [29], Haywood *et al.* (2005) [30], Martin and Martin (2003) [31].
Commentary	Marques *et al.* (2014) [32], Olde-Rikkert *et al.* (2013) [8], Philp (1997) [3], Philp *et al.* (2001) [33], Philp (2000) [34], Richardson (2001) [7].

^a^Signifies same sample population for Lambert.

^b^Signifies same sample population for Melis.

^c^Signifies same sample population for van Kempen.

Fourteen peer-reviewed articles presenting empirical data were assessed by three researchers using the relevant Critical Appraisal Skills Programme (CASP) checklists to assess the methods and whether biases and confounders had been appropriately adjusted for. Through an internal discussion between three reviewers (C.C., N.C. and G.S.), a consensus was reached of nine papers which were assessed to be of acceptable quality, summarised in Table 2. Three papers (from one study) included the use of EASY-Care administered in hospital with patients no longer receiving consultant-led care [9–11]. The sample was predominantly from community-dwelling older people, so they were included in the review.

**Table 2.** Summary of peer-reviewed empirical data of acceptable quality

Author	Study design	Country	Population	Setting	Sample size	Paper contributes towards evidence for:		
						Acceptability	Reliability	Validity
Keiren *et al.* [17]	Feasibility study (mixed methods)	Netherlands	Family practitioners Primary care nurses (PCNs) Older people	General practice	Professionals = 25 Older people = 9	✓		
Lambert *et al.* [10]	Cross-sectional study	UK	Older people	Nursing homes, Residential care, hospitals	119 Older people	✓		
Lambert *et al.* [9]	Cross-sectional study	UK	Older people	Nursing homes, Residential care, hospitals	119 Older people	✓		
Lambert *et al.* [11]	Cross-sectional study	UK	Older people	Nursing homes, Residential care, hospitals	119 Older people	✓		✓
Melis *et al.* [19]	Randomised control trial	Netherlands	Vulnerable older adults at home	Community	151 Older people (Intervention = 85 Control = 66)	✓		
Melis *et al.* [18]	Randomised control trial	Netherlands	Vulnerable older adults at home	Community	151 Older people (Intervention = 85 Control = 66)	✓		
Philip *et al.* [21]	Cross-sectional study (mixed methods)	Colombia, Kerala, Lesotho, UK, Tonga, Iran	Older people, Clinicians	Primary care, Community, Secondary care	Older people = 115 Clinicians = 79	✓		
van Kempen *et al.* [26]	Qualitative observational study	Netherlands	Frail older adults, family practitioners, PCNs	Primary care	Older people = 161 Professionals = 18	✓		
van Kempen *et al.* [27]	Validation study	Netherlands	Frail older adults, family practitioners, PCNs	6 GP practices	587 older people		✓	✓

## Overview of included studies

Table 1 details the studies included in this review and the different types of publication. This includes empirical evidence, narrative (non-systematic) reviews of the literature and commentaries documenting the evolution of EASY-Care. The empirical evidence was based on a total sample of 2,176 (range 9–587 per study) patients or older people (age range 57–99 years) and 421 (range 9–298 per study) practitioners. These studies were also based around a wide geographical distribution with sites in the United Kingdom, Netherlands, Russia, Portugal, Albania, Kosovo, Tanzania, Columbia, Iran, India and Tonga.

## Data extraction and synthesis

Data extraction was undertaken based on the CASP quality assessment tool to extract information from included articles in relation to study participants/sample, type of interventions using EASY-Care, comparator tools or standards, assessment outcomes and measurements of reliability, validity and acceptability. Due to the diversity of articles included in the review (e.g. commentaries, narrative reviews, randomised controlled trials, qualitative studies), a meta-analysis was not feasible. Therefore, a narrative synthesis of data relating to the validity, reliability and acceptability of EASY-Care was undertaken.

### Validity—personal and population-level needs assessment

Validity refers to how effective a tool is at measuring what it is intended to measure [35]. There is a wealth of knowledge concerning the face, content, criterion and cross-cultural validity of EASY-Care, but more studies need to be done to establish concurrent and convergent validity. EASY-Care bases itself largely on the aforementioned previously validated tools from which it assumes external validity [7]. Professional geriatricians have contributed to the content, enhancing face and content validity [8]. When the tool was compared against other gold standard health measurements from a population of 50 patients, there were mixed Cohen's Kappa values (range 0.39–1) [22]. Good intra-class correlations for loneliness, morale and the disability score gave evidence of criterion validity [22]. The successful linkage of 63 of the 75 questions (of the Portuguese EASY-Care version) to domains of the WHO International Classification of Functioning, Disability and Health (ICF) further increases the content validity of EASY-Care [32]. Cross-cultural validation is evident affirming EASY-Care's use internationally [16, 21].

### Reliability—personal and population-level needs assessment

Reliability of a tool refers to how consistent the results are when collected multiple times and to how much variance in the measure is due to chance. Evidence of reliability of EASY-Care as a needs assessment tool is limited to one article. From the same population of 50 patients, different assessors undertaking assessments in a 2-week test–retest period yielded generally positive kappa values ranging from −0.06 to 0.82 [22]. The domains scoring poorly (communication, feeding, telephone use and cognitive impairment) were explained by having poor spread of data, with the authors admitting further testing was required. Literature as recent as 2013 [8] cites these figures, suggesting no other reliability assessments of EASY-Care as a needs assessment or population-level data tool have been published. No evidence of internal consistency is published in English.

### Validity—diagnostic tool

EASY-Care has also been used as a clinical decision support tool, the EASY-Care Two Step Older Persons Screening method (EASY-Care TOS), for which validation studies have been undertaken. Concurrent validity was studied when a comparison of EASY-Care against the Fried Frailty Criteria and the Rockwood Frailty Index produced correlation coefficients of 0.52 (*P* < 0.001) and 0.63 (*P* < 0.001), respectively, when administered on the same population of 587 older people [27]. Convergent validity was calculated when EASY-Care was compared against two other frailty measurement tools, correlating significantly with the Fried Frailty Criteria and the Rockwood Frailty Index producing Spearman Rho statistics of 0.458 and 0.573 (both *P*-values < 0.001), respectively [25]. It is important to show good predictive validity in any diagnostic tool. Using the EASY-Care TOS method as a predictor of functional decline and an indicator of frailty resulted in greater predictive value than objective patient measurements. This was mainly because this method makes use of the GP's prior knowledge of the patient [24].

### Reliability—diagnostic tool

A small sample of 19 from those included in the EASY-Care TOS study showed promising signs of reliability, with an 89% agreement between assessors and a test–retest kappa value of 0.63 with no significant differences [27]. However, the authors offer a forewarning of the reliability of using the EASY-Care TOS method because of the subjectivity associated with each GP's decision making process.

### Acceptability of EASY-Care

EASY-Care is available in both paper and electronic format. Trialling of the electronic version was piloted in the United Kingdom in 2004 [28], but no results from this testing have been forthcoming. EASY-Care is shown to be a highly usable tool in community and residential groups internationally. Older people and assessors testify to its feasibility with a small minority expressing difficulties in using EASY-Care, both as a needs assessment and diagnostic test [9, 21, 23]. The developers suggest reasonable re-wording or re-phrasing to be more consistent with culturally appropriate dialogue. Assessor feedback also helps to improve EASY-Care's development.

Having received international acclaim for the simplicity of the language [21], older people can reasonably be expected to self-complete or complete with the assistance of family or friends. However, nurses are deemed the most appropriate to assist in completing the assessment. The inter-personal skills synonymous with nursing staff as skilled assessors [7], their ability to build a rapport with patients (especially when asking potentially sensitive questions) [10] and the option to take on the assessment from time-constrained GPs [17] are all prime examples of this. They also would require less training than voluntary assessors and may be more objective in their assessment, given the tendency of carers and older people to over or under report the levels of dependency [36].

The implementation of an EASY-Care-based intervention within a complex intervention (Dutch Geriatric Intervention Programme) has been shown to improve patient quality of life in a cost-effective manner with a significantly greater proportion of successfully treated patients in the intervention arm, at a willingness to pay of €34,000 [18].

## Discussion

This review has found strong evidence of the acceptability of EASY-Care when used as a tool to assess personal needs, with high levels of feasibility and usability, and some evidence of cost-effectiveness. There is reasonable evidence of validity through the inclusion of validated scales in the tool, the contribution of professional geriatricians to the content and good correlation with other health measurement tools. Additionally, it maps well onto the WHO ICF classifications and has evidence of cross-cultural validity. The one study assessing reliability had small sample sizes and a poor spread of data. Only minimal evidence was found for the use of the EASY-Care tool for population-level needs assessments and as a diagnostic tool for frailty. Also, there was little evidence regarding the use of EASY-Care in practice.

Due to our systematic and thorough search of the literature, we feel our findings are an accurate representation of the evidence base. However, only articles published in English were considered. Seven foreign language articles (German, French, Dutch, Portuguese and Polish) were not reviewed and they may hold valuable data relevant to this review. EASY-Care's headquarters are based in the United Kingdom where an annual conference is held, where it is reasonable to expect significant findings to be reported. Several papers included in this review have been conducted in countries where English is not the first language but have still reported back in English so we do not expect our limitation to English language articles to introduce bias in the findings reported here.

Considering that EASY-Care was accredited for use in the Single Assessment Procedure (SAP) in England in 2001, we expected to find in the literature more examples of the use of EASY-Care in practice. Due to limited evidence outside of a research context, we are unable to say whether EASY-Care is useful in practice or not. However, there is evidence that it is preferred over other accredited tools [11].

There is good evidence available for care providers across the world to consider using EASY-Care for assessing community need and as a frailty diagnostic tool. More research documenting the use of EASY-Care in practice would be helpful, building on evidence from the Netherlands that a geriatric intervention programme with EASY-Care at its core can produce better health outcomes in an effective and cost-effective manner [18, 19]. Results from the acceptability of using the electronic format would also be informative to implementers of comprehensive geriatric assessments.

From a commissioning perspective, using EASY-Care as a standardised needs assessment tool can provide population-level data, thus assisting in health and social care planning [12]. Practitioners should take in to consideration what supplementary financial, staffing and medical resources are required to successfully undertake an EASY-Care assessment. Low-income countries using EASY-Care may be happy to use the tool but may not be able to cater to their citizens needs identified due to lack of resources [21]. This may not necessarily be a negative as it can help guide the efficient use of resources when they are scarce. The use of EASY-Care in an electronic format could also facilitate better health and social care planning for individuals should the infrastructure to share patient information be available, thus eliminating potential duplication of questioning and a more integrated system. When considering other needs assessment tools, the reviews of comprehensive geriatric assessment tools included in this review are all at least 10 years old. We are currently undertaking an updated review of CGAs to aid practitioners, commissioners and service providers in choosing an appropriate tool.

The potential benefits of using EASY-Care in practice are described [34] with a good fit to nursing practice either as a needs assessment tool [11] or as a diagnostic tool [17]. Importantly, these positive reports that justify the benefits of using EASY-Care in practice are internationally recognised, with consistent responses from participants in different countries [16, 21]. Overall, EASY-Care is a valid, comprehensive and acceptable tool centred around the older person's priorities for promoting their own well-being. However, the lack of current reliability testing suggests that further research is warranted.

## Conclusion

This systematic review provides a comprehensive summary of the available evidence for the EASY-Care assessment tool for different purposes. While the literature reports favourably on the validity and acceptability of EASY-Care as a personal needs assessment tool, there is limited evidence for reliability and for its use as a population-level needs assessment or diagnostic tool for frailty. Therefore, it is concluded that further research is required to test the reliability of the tool and the validity and reliability for different applications. The lack of evidence of this SAP accredited tool highlights the need for further study assessing the impact of EASY-Care in routine practice.

**Key points**

- There is a lack of reliability evidence for EASY-Care, with further testing required.
- There is strong evidence for the validity and acceptability of EASY-Care as a personal needs assessment and good acceptability internationally.
- Evidence suggests that an EASY-Care-based intervention can have beneficial health outcomes.

## Conflicts of interest

None declared.

## Funding

This paper presents independent research commissioned by the National Institute for Health Research (NIHR) as part of the Collaboration for Leadership in Applied Health Research and Care East Midlands (CLAHRC EM). The views expressed are those of the authors and not necessarily those of the NHS, the NIHR or the Department of Health.

## Supplementary data

Supplementary data mentioned in the text are available to subscribers in *Age and Ageing* online.

Supplementary Data
