# Supplementary material for: Systematic review of EASY-care needs assessment for community-dwelling older people
Source: Age Ageing. 2015 Apr 24;44(4):559–65. doi: 10.1093/ageing/afv050 (PMC4476847; doi:10.1093/ageing/afv050)
Supplement: Supplementary Data [file supp_44_4_559__index.html]

Systematic review of EASY-care needs assessment for community-dwelling older people — Supplementary Data 

# Systematic review of EASY-care needs assessment for community-dwelling older people

## Supplementary Data

Supplementary Data

**Files in this Data Supplement:**

- Supplementary Data - Docx file
