# Supplementary material for: Systematic review of EASY-care needs assessment for community-dwelling older people
Source: Age Ageing. 2015 Apr 24;44(4):559–65. doi: 10.1093/ageing/afv050 (PMC4476847; doi:10.1093/ageing/afv050)
Supplement: Supplementary Data [file supp_afv050_afv050supp.docx]

## REFERENCES (Please note: The very long list of references supporting this review has meant that only the most important are listed here and are represented by bold type throughout the text. The full list of references is available on the journal website).

1. Beswick AD, Rees K, Dieppe P, Ayis S, Gooberman-Hill R, Horwood J, et al. Complex interventions to improve physical function and maintain independent living in elderly people: a systematic review and meta-analysis. The Lancet.371(9614):725-35.

2. Clegg A, Young J, Iliffe S, Rikkert MO, Rockwood K. Frailty in elderly people. The Lancet. 2013;381(9868):752-62.

3. Philp I. Can a medical and social assessment be combined? Journal of the Royal Society of Medicine. 1997;90 Suppl 32:11-3.

4. Mahoney FI, Barthel DW. Functional Evaluation: The Barthel Index. Md State Med J. 1965 Feb;14:61-5.

5. Fillenbaum GG, Smyer MA. The development, validity, and reliability of the OARS multidimensional functional assessment questionnaire. Journal of gerontology. 1981 Jul;36(4):428-34.

6. EASYCare Group. EASYCare tool website (<http://www.easycareproject.org/)>. 2014.

7. Richardson J. The Easy-Care assessment system and its appropriateness for older people. Nurs Older People. 2001;13(7):17-9.

8. Olde-Rikkert MG, Long JF, Philp I. Development and evidence base of a new efficient assessment instrument for international use by nurses in community settings with older people. International Journal of Nursing Studies. [Research Support, Non-U.S. Gov't

Validation Studies]. 2013 Sep;50(9):1180-3.

9. Lambert S, Gardner L, Thomas V, Davies S. Assessing older people with complex care needs using EASYCare a pre defined assessment tool. Research, Policy and Planning. 2007;25(1):43-56.

10. Lambert S, Thomas V, Gardner L. 'Introducing yourself to strangers': nurses' views on assessing older people with complex care needs. Journal of Research in Nursing. 2007;12(4):349-61.

11. Lambert S, Cheung W, Davies S, Gardner L, Thomas V. Comparison of two tools developed to assess the needs of older people with complex care needs. Journal of Research in Nursing. 2009;14(5):421-36.

12. Bath P, Philp I, Boydell L. Standardized health check data from community-dwelling elderly people: the potential for comparing populations and estimating need. Health Soc Care Community. 2000;8(1):17-21.

13. Bath P, Philp I. A Hierarchical classification of dependancy amongst older people: use of artificial neural networks. Health Care in Later Life. 1998;3(1):59-69.

14. Faculty of Higher Nursing Education of the Sechenov Medical Academy. Evaluation of EASY-Care Single Assessment tool in Moscow [Unpublished]. 2008.

15. Fernandes L, Gonalves-Pereira M, Leuschner A, Martins S, Sobral M, Azevedo LF, et al. Validation study of the Camberwell Assessment of Need for the Elderly (CANE) in Portugal. Int Psychogeriatr. 2009;21(1):94-102.

16. Jerilu N, Toçi E, Burazeri G, Ramadani N, Philp I, Brand H. Cross-cultural adaptation of an instrument measuring older people health needs and priorities in Albania and Kosovo. Albanian Medical Journal. 2013;2:109-14.

17. Keiren SM, Kempen JA, Schers HJ, Olde Rikkert MG, Perry M, Melis RJ. Feasibility evaluation of a stepped procedure to identify community-dwelling frail older people in general practice. A mixed methods study. European Journal of General Practice. 2013.

18. Melis RJ, Adang E, Teerenstra S, van Eijken MI, Wimo A, van Achterberg T, et al. Cost-effectiveness of a multidisciplinary intervention model for community-dwelling frail older people. J Gerontol A Biol Sci Med Sci. 2008 Mar;63(3):275-82.

19. Melis R, Van Eijken M, Teerenstra S, Van Achterberg T, Parker S, Borm G, et al. A randomized study of a multidisciplinary program to intervene on geriatric syndromes in vulnerable older people who live at home (Dutch EASYcare Study). J Gerontol Ser A Biol Sci Med Sci. 2008 March;63(3):283-90.

20. Msambichaka B, Urassa H, Wiliam J, Shemdoe, Mkopi A, Dillip A, et al. Evaluation of the EASY-Care Tool in the Tanzanian context. (Unpublished). 2014.

21. Philip KE, Alizad V, Oates A, Donkin DB, Pitsillides C, Syddall SP, et al. Development of EASY-Care, for Brief Standardized Assessment of the Health and Care Needs of Older People; With Latest Information About Cross-National Acceptability. J Am Med Dir Assoc. 2014;15(1):42-6.

22. Philp I, Lowles RV, Armstrong GK, Whitehead C. Repeatability of standardized tests of functional impairment and well-being in older people in a rehabilitation setting. Disability and Rehabilitation. 2002 Mar;24(5):243-9.

23. Philp I, Newton P, McKee KJ, Dixon S, Rowse G, Bath P. Geriatric assessment in primary care: formulating best practice. British journal of community nursing. 2001;6(6):290-5.

24. van Kempen J. The identification of frail older persons in primary care: the development and validation of the EASY-Care Two step Older persons Screening. [PhD Thesis]. 2013.

25. Van Kempen J, Schers H, Melis R, Olde Rikkert M. Convergent validity of a two-step identification method for frailty in older persons: Easycare-TOS. Eur Geriatr Med. [Conference Abstract]. 2012 September;3:S60.

26. van Kempen JA, Schers HJ, Jacobs A, Zuidema SU, Ruikes F, Robben SH, et al. Development of an instrument for the identification of frail older people as a target population for integrated care. British Journal of General Practice. [Evaluation Studies Observational Study Research Support, Non-U.S. Gov't]. 2013 Mar;63(608):e225-31.

27. van Kempen JA, Schers HJ, Melis RJ, Olde Rikkert MG. Construct validity and reliability of a two-step tool for the identification of frail older people in primary care. J Clin Epidemiol. [Research Support, Non-U.S. Gov't]. 2014 Feb;67(2):176-83.

28. Foreman P, Thomas S, Gardner I. The review and identification of an existing, validated,comprehensive assessment tool. 2004.

29. Haywood K, Garratt A, Schmidt L, Mackintosh A, Fitzpatrick R. Health Status and Quality of Life in Older People - A Structured Review of Patient-reported Health Instruments. Report from the Patient-reported Health Instruments Group (formerly the Patient-assessed Health Outcomes Programme) to the Department of Health, April 2004.

30. Haywood KL, Garratt AM, Fitzpatrick R. Older people specific health status and quality of life: a structured review of self-assessed instruments. Journal of Evaluation in Clinical Practice. 2005 Aug;11(4):315-27.

31. Martin GJO, Martin IR. Assessment of Community Dwelling older people in New Zealand: A review of the tools. 2003.

32. Marques A, Martins A, Jacome C, Figueiredo D. Linking the EASY-Care Standard to the International Classification of Functioning, Disability and Health. Disability and Rehabilitation. 2014;36(7):593-9.

33. Philp I, Lowles RV. Simple measures for assessing the physical mental and social functioning of older people. Journal of the British Society of Gerontology. 2001.

34. Philp I. A systematic approach to the assessment of older people. Geriatric Medicine. 2000;30(5):15-9.

35. Streiner DL, Norman GR. Health Measurement Scales: A practical guide to their development and use. 4 ed. Oxford: Oxford University Press; 2008.

36. Ostbye T, Tyas S, McDowell I, Koval J. Reported activities of daily living: agreement between elderly subjects with and without dementia and their caregivers. Age Ageing. 1997 Mar;26(2):99-106.
